# High Performance Polymer/Ionic Liquid Thermoplastic Solid Electrolyte Prepared by Solvent Free Processing for Solid State Lithium Metal Batteries

**DOI:** 10.3390/membranes8030055

**Published:** 2018-08-02

**Authors:** Francisco González, Pilar Tiemblo, Nuria García, Oihane Garcia-Calvo, Elisabetta Fedeli, Andriy Kvasha, Idoia Urdampilleta

**Affiliations:** 1 Instituto de Ciencia y Tecnología de Polímeros, ICTP-CSIC, Juan de la Cierva 3, 28006 Madrid, Spain; fgonzalez@ictp.csic.es (F.G.); ngarcia@ictp.csic.es (N.G.); 2 CIDETEC Energy Storage, Parque Científico y Tecnológico de Gipuzkoa, Paseo Miramón 196, 20009 Donostia-San Sebastián, Spain; ogarcia@cidetec.es (O.G.-C.); efedeli@cidetec.es (E.F.); iurdampilleta@cidetec.es (I.U.)

**Keywords:** solid state battery, thermoplastic polymer electrolyte, ionic liquid, sepiolite, inorganic filler

## Abstract

A polymer/ionic liquid thermoplastic solid electrolyte based on poly(ethylene oxide) (PEO), modified sepiolite (TPGS-S), lithium bis(trifluoromethanesulfonyl)imide (LiTFSI), and 1-Butyl-1-methylpyrrolidinium bis(trifluoromethanesulfonyl)imide (PYR_14_TFSI) ionic liquid is prepared using solvent free extrusion method. Its physical-chemical, electrical, and electrochemical properties are comprehensively studied. The investigated solid electrolyte demonstrates high ionic conductivity together with excellent compatibility with lithium metal electrode. Finally, truly Li-LiFePO_4_ solid state coin cell with the developed thermoplastic solid electrolyte demonstrates promising electrochemical performance during cycling under 0.2 C/0.5 C protocol at 60 °C.

## 1. Introduction

Highly efficient, light, safe, and long-lasting rechargeable batteries are the goal of all the researchers and producers involved in the energy storage business. So far, lithium ion batteries (LIBs) represent the most promising answer; however, the booming growth of demand spotlighted the drawbacks of such technology. The major intrinsic limitation of LIBs is the low theoretical specific capacity (372 mAh·g^−1^) of the traditional graphite anode, which does not allow the increase of practical LIB energy density to more than 300 Wh·kg^−1^. Lithium metal represents the best alternative anode material to produce high energy density batteries because it possesses the lowest standard potential (E_o_ = −3.04 V versus standard hydrogen electrode) and the highest theoretical capacity (3.860 mAh^−1^) [1]. Unfortunately, this technology is not ideal and presents several issues such as dendrite growth, instability of lithium metal with the most part of classical organic liquid electrolytes, low coulombic efficiency, poor cyclability, and poor safety due to leakage and high flammability of the liquid electrolyte based on a mixture of carbonate solvents [2,3,4].

Solid polymer electrolytes (SPEs) are without a doubt among the key solutions to overcome such limitations toward high energy density, efficient, and safe solid state batteries (SSB) [4,5,6,7]. Indeed, these solid ion conductive membranes can replace microporous separators impregnated by volatile flammable organic electrolytes [5,6], acting as physical barrier against dendrite growth reducing the possibility of short-circuit, thermal runaway, and explosion, significantly improving the safety of the battery [8,9,10]. However, poor ionic conductivity at room temperature due to low mobility of the lithium cations in the solid polymer matrix, and the loss of mechanical properties in the conductive molten state at higher temperature, limit their spread in the battery market [11]. Several solutions have been proposed to increase ionic conductivity while maintaining good mechanical properties [12,13]. Many of them are based on the addition of low molecular weight compounds with adequate electrochemical properties coupled to the creation of physical or chemical crosslinking sites at the polymer [14]. The employment of inorganic fillers and the introduction of sufficient amount of low molecular weight compounds are listed among the most relevant examples. Adding inorganic fillers proved to favor the performance of the battery by (i) preventing crystallization by hindering the supramolecular arrangement of the polymer chains; (ii) favoring ionic dissociation, improving the matrix/solid electrolyte interface (SEI) interaction thanks to the contribution of different possible surface groups; and (iii) increasing mechanical resistance and stability [15,16,17]. The employment of low molecular weight compounds also proved to be an effective measure to enhance the electrochemical performance of a solid state battery [18]. Among them, and despite some drawbacks (high cost and some instability at lithium deposition potential [19]), room temperature ionic liquids (RTILs) are, probably, the most promising materials thanks to their negligible vapor pressure, low flammability, high ionic conductivity in comparison with solid polymer electrolytes, and their ability to form an effective solid electrolyte interphase onto the lithium metal electrode surface [20,21,22]. Several recent studies demonstrated that the presence of RTILs can enhance significantly the electrochemical properties of the solid state battery, such as, for instance, improving the long-time stability in the stripping/plating from lithium metal electrodes [23,24]. Furthermore, it has recently been demonstrated that free RTILs in a polymeric solid matrix can undergo percolation, creating a highly conductive pathway across the electrolyte and a wet interfacial layer that greatly improves the interfacial compatibility with the electrodes [25].

On the other hand, the always increasing demand of electronic devices goes together with a growing concern about a sustainable future and cost considerations. The combination of these two factors bursts the research toward the development of green processes to obtain high performance materials. In this optic, fast production methods that employ recyclable materials and reduce or eliminate completely the use of harmful organic solvents are the main goal of all the efforts. Thermoplastic polymers represent a valid option to develop solid electrolytes because they can be processed easily by extrusion and shaped by hot-pressing or lamination, none of which require solvent, and can be theoretically recycled and reprocessed, in this way reducing the final cost of solid state batteries, which is crucial for their implementation in the market [26,27,28].

In this context, this work presents the solvent-free preparation of a thermoplastic polymer electrolyte (TPE) consisting on a polymeric matrix, ad hoc modified inorganic fillers, an ionic liquid, and a lithium salt. More precisely, the TPE is composed by poly(ethylene oxide) (PEO), surface modified sepiolite (TPGS-S), lithium bis(trifluoromethanesulfonyl)imide (LiTFSI), and 1-Butyl-1-methylpyrrolidinium bis(trifluoromethanesulfonyl)imide (PYR_14_TFSI), prepared by solvent-free extrusion method. This TPE is compared with a well-studied reference electrolyte consisting of PEO and LiTFSI [29]. The extensive physical and electrochemical characterization of the new TPE is presented in this article. The developed solid electrolyte demonstrated high ionic conductivity, good electrochemical stability, excellent compatibility with lithium metal, and promising cycling performance in truly solid state Li-LiFePO_4_ coin cell prototype.

## 2. Materials and Methods 

### 2.1. Reagents

For the preparation of the solid electrolytes, the following materials were used: PEO: Mn 5 × 10^6^ g·mol^−1^ for the TPE, Mn 6 × 10^5^ g·mol^−1^ for the reference electrolyte, and Mn 4 × 10^5^ g·mol^−1^ for the positive electrode preparation, all purchased from Sigma-Aldrich (St. Louis, MO, USA). d-α-tocopherol polyethylene glycol 1000 succinate (TPGS), used to prepare the modified sepiolite (TPGS-S), was purchased from Sigma-Aldrich and used as received. Details on the preparation of the TPGS-S have appeared elsewhere [30]. Battery grade LiTFSI and PYR_14_TFSI with 99.9% of purity were purchased from Solvionic (Toulouse, France). Dry acetonitrile with 99.8% of purity was purchased from Scharlab (Barcelona, Spain). All the reagents were stored in dry room with dew point below −50 °C; they were used without further purification.

### 2.2. Synthesis and Preparation of Materials

Reference solid polymer electrolyte (PEO-LiTFSI) was prepared as follows: LiTFSI was dissolved in acetonitrile and stirred with a mechanical stirrer for 30 min. PEO, Mn 6 × 10^5^ g·mol^−1^, was slowly added and the mixture was stirred for 5 h to guarantee the complete solubilization of all reagents. The molar ratio of EO/Li was chosen to be 20. The amount of solid in the acetonitrile solution was set to 12 wt %. Self-standing membranes of reference PEO-LiTFSI electrolyte were obtained by solvent casting over Teflon sheets. The casted solution was dried for 2 h at 35 °C and then for 17 h at 60 °C under reduced pressure. PEO-LiTFSI electrolyte formulation is given in Table 1.

TPE was prepared in accordance with method reported earlier [26]. Briefly, all components were physically premixed and then melt compounded in a Haake MiniLab extruder (Haake Minilab, Thermo Fisher Scientific, Waltham, MA, USA). Processing was carried out at a shear rate of 80 rpm during 20 min and at 160 °C. Afterwards, TPE extrudate was processed by hot pressing at 75 °C. TPE electrolyte formulation is given in Table 1.

### 2.3. Physicochemical Characterization

Characterization of electrolytes was done on films of controlled thickness processed by compression molding at 75 °C during 3 min.

Scanning electron microscopy (SEM) was performed with a Hitachi SU-8000 (Hitachi Ltd., Tokyo, Japan). Samples were fractured after immersion in liquid nitrogen and the sections were observed unmetalized.

Differential scanning calorimetry (DSC) studies were performed in a TA Instruments Q100 (TA Instruments, New Castle, DE, USA). The heat flow was recorded as follows: two cooling-heating cycles at 10 °C·min^−1^ from 120 °C to −80 °C, followed by a second cooling-heating cycle from 120 °C to −80 °C at 20 °C·min^−1^. DSC data included in Table 1 were obtained from the second DSC heating trace at 10 °C·min^−1^. The crystallinity percentage (χ_c_) was determined considering 100% crystalline PEO heat of melting as Δ*H*_m_ = 197 J·g^−1^ [31]. The % χ_c_ in Table 1 is referred to the weight of the electrolyte and not to the weight fraction of PEO.

Thermogravimetric analysis (TGA) was performed in a TA Q-500 in nitrogen atmosphere at 10 °C·min^−1^ up to 800 °C.

Determination of diffusion coefficients (*D*) was done by ^7^Li and ^19^F pulsed field gradient-NMR (PFG-NMR) in a Bruker AvanceTM 400 spectrometer (Bruker BioSpin GmbH, Rheinstetten, Germany) as reported before [26]. The lithium transference number measured by NMR ($t_{{\mathrm{Li}}^{+}}^{\mathrm{NMR}}$) was calculated using Equation (1). It was not possible to measure *D* of the cation (*D*_Pyr_), because of the overlapping with PEO protons, so it was estimated to be about 10% lower than TFSI, according to bibliographic data [32].

(1) $$t_{{\mathrm{Li}}^{+}}^{\mathrm{NMR}}=\frac{D_{\mathrm{Li}}c_{\mathrm{Li}}}{D_{\mathrm{Li}}c_{\mathrm{Li}}+D_{\mathrm{TFSI}}c_{\mathrm{TFSI}}+D_{\mathrm{Pyr}}c_{\mathrm{Pyr}}}$$

Creep experiments were done as follows: electrolyte films of about 500 μm were sandwiched between two gold electrodes of 20 mm of diameter, and placed on a heating plate with a 0.5 kg load on top and kept 20 min at 70 °C, followed by 20 min at 90 °C.

### 2.4. Electrochemical Characterization

The ionic conductivity of the TPE and PEO-LiTFSI electrolytes was determined by electrochemical impedance in a NOVOCONTROL GmbH Concept 40 broadband dielectric spectrometer (Novocontrol Technologies GmbH, Montabaur, Germany) in the temperature range of 50 °C to 90 °C and in the frequency range between 0.1 Hz and 10^7^ Hz. Disk films of dimensions of 2 cm diameter and ~500 μm thickness were inserted between two gold-plated flat electrodes, then a frequency sweep was done every 10 °C, cooling to −50 °C and then heating to 90 °C; thereafter, the same measurements were done but cooling from 85 °C to 25 °C. Ionic conductivity was calculated by using conventional methods based on the Nyquist diagram and the phase angle as a function of the frequency plot. The values that appear in Table 1 correspond to the second heating measurement.

Lithium transference number ($t_{{\mathrm{Li}}^{+}}$) of the TPE was obtained at 60 °C by combined alternating current (AC) impedance and direct current (DC) polarization measurements using a Solartron Analytical 1400 CellTest System (cell test, City, UK) coupled with frequency response analyzer 1455 (Ametek) of a symmetrical solid state Li/TPE/Li coin cell (2025, Hohsen, City, Japan). Coin cells were prepared using high-purity lithium metal foil (Albermale, Charlotte, NC, USA) with thickness of 50 μm. Before the measurement, the assembled coin cells were kept at 60 °C overnight to achieve a good contact and stable interface between the solid electrolyte and lithium metal electrodes. Successively, a DC potential (Δ*V* = 5 mV) was applied until a steady current was obtained; then, initial (*I*_o_, after 5 milliseconds) and steady state (*I*_ss_) currents that flow through the cell were measured. Impedance spectra were recorded (from 1 MHz to 1 Hz) with 10 mV sinusoidal amplitude before and after DC polarization. Subsequently, initial (*R*_o_) and final (*R*_ss_) bulk resistances of the electrolyte, and initial (*R*_Co_) and final (*R*_Css_) charge transfer resistances (Ω) of the interfacial layers Li metal electrode/electrolyte were derived from electrochemical impedance spectra using ZView software 3.5 (Scribner, Southern Pines, NC, USA) Using these measured values, $t_{{\mathrm{Li}}^{+}}$ was calculated by the following Equation (2) [33,34].

(2) $$t_{{\mathrm{Li}}^{+}}=\frac{I_{\mathrm{ss}}\cdot R_{\mathrm{ss}}\cdot \left(\Delta V-I_{o}\cdot R_{\mathrm{Co}}\right)}{I_{o}\cdot R_{o}\cdot \left(\Delta V-I_{\mathrm{ss}}\cdot R_{\mathrm{Css}}\right)},$$

The electrochemical stability window of the TPE was evaluated in three-electrode cells using a Solartron Analytical 1400 CellTest System (Ametek) coupled with a frequency response analyzer 1455 (Ametek). To do so, a solid-state three electrode cell (HS-3E, Hohsen), using stainless steel as a working electrode, a lithium metal (50 µm) disc as a counter electrode, a lithium metal ring as a reference electrode, and a solid electrolyte membrane (80–100 µm) placed between electrodes was fabricated. The cyclic voltammetry (CV) test was carried out at a linear scan rate of 1 mV·s^−1^ to determine the electrochemical performance in cathodic (from OCV to −0.5 V) range. The oxidation stability of the investigated solid electrolyte was determined by linear sweep voltammetry (LSV) from OCV to 6 V at a scan rate of 1 mV·s^−1^. Both CV and LSV experiments were performed at 60 °C using different TPE samples.

Galvanostatic stripping-plating studies were carried out at 60 °C in a symmetrical Li/TPE/Li coin cell (2025, Hohsen), using two lithium metal discs (Albermale, high-purity foil, 50 µm) and TPE films (80–100 µm) placed in between. The measurements were performed with the help of BaSyTec cell test system (BaSyTec, Asselfingen, Germany) at 60 °C. Galvanostatic cycles were run by applying symmetrical 1 mA·cm^−2^ current for 2 h with depth of cycling of 2 mAh·cm^−2^.

Galvanostatic charge-discharge test in solid-state coin cells with lithium metal anode (Albermale, high-purity foil, 50 µm) and composite LiFePO_4_ (LFP) cathode was performed at 60 °C using the BaSyTec cell test system. The cathode consisted of micro-scale carbon coated LFP material (D50: 2–4 μm), PEO-LiTFSI solid electrolyte (EO/Li~20) as ionic conductive binder, and carbon black as a conductive additive. Superficial capacity of the prepared positive electrode was 0.5 mAh·cm^−2^. A carbon coated aluminum current collector was used to enhance interfacial resistance and avoid aluminum corrosion in the presence of TFSI anions. Solid-state coin cells were assembled in a dry room with dew point below −50 °C. Once assembled, the cells were kept for 3 h at 60 °C and then cycled within the 2.5–3.8 V range at the same temperature using BaSyTec cell test system. It is important to note that cell design, assembly, and formation procedures were not optimized in this study.

## 3. Results

### 3.1. Physicochemical Investigation

**Figure 1:**
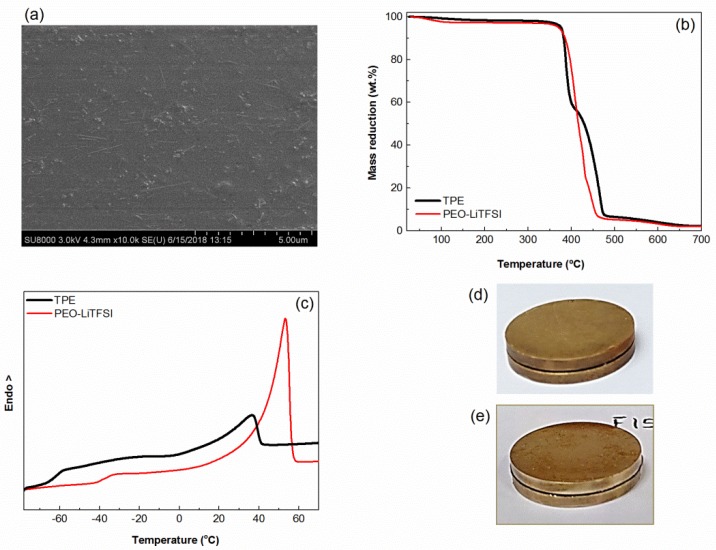
(**a**) Scanning electron microscopy (SEM) image of the thermoplastic polymer electrolyte (TPE) (cross-section); (**b**) thermogravimetric analysis (TGA) of the poly(ethylene oxide) (PEO)-lithium bis(trifluoromethanesulfonyl)imide (LiTFSI) and TPE; (**c**) differential scanning calorimetry (DSC) traces of the PEO-LiTFSI and TPE; and (**d**) pictures showing the electrolyte appearance after the creep test (see text for details).

Similar TPE reported before [23,25] have shown two properties that make them interesting candidates as electrolytes, their liquid nature at the microscopic scale and their ability to remain as solids at the macroscale up to 90 °C for long periods of time [23]. Figure 1 summarizes the physicochemical characterization performed with both the TPE under study and the reference PEO-LiTFSI, which includes a SEM micrograph of the TPE, and the TGA, DSC, and creep experiments of both electrolytes. 

First of all, Figure 1a shows the excellent dispersion of the sepiolite nanofibers in the electrolyte. Figure 1b shows that the thermal stabilities of the TPE and PEO-LiTFSI in nitrogen are very similar and will favor the overall solid-state battery safety. Figure 1c shows how the TPE has two well defined transitions, the PEO glass transition close to −60 °C and a melting endotherm slightly under 40 °C caused by the scarce crystalline phase in the TPE. On its turn, PEO-LiTFSI has a T_g_ at about −38 °C and a melting endotherm at 50 °C, the latter caused by the crystalline PEO phase, which amounts to 31%. Both the higher T_g_ and the higher crystalline fraction of PEO-LiTFSI make this electrolyte more rigid than TPE. Figure 1d and 1e show the appearance of the sandwiches (electrode-electrolyte) of PEO-LiTFSI and TPE, respectively, after the creep tests. No creep is seen in either sample after being subjected to the temperature cycles under pressure.

The ionic diffusivity in the TPE has been obtained by PFG-NMR experiments at 25 °C, and values are in the range of those obtained for similar TPE [23]: *D*_Li_ = 0.6 × 10^−12^ m^2^⋅s^−1^ and *D*_TFSI_ = 3.9 × 10^−12^ m^2^⋅s^−1^. A transport number $t_{{\mathrm{Li}}^{+}}$ = 0.03 at 25 °C can be estimated from these diffusion coefficients using Equation (1).

### 3.2. Electrochemical Investigation

**Figure 2:**
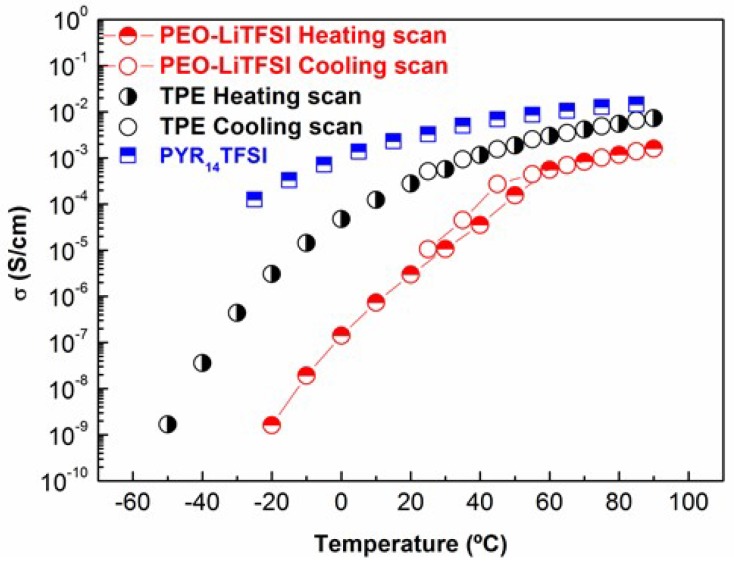
Ionic conductivity of the TPE and PEO-LiTFSI on heating and cooling scans. Ionic conductivity of 1-Butyl-1-methylpyrrolidinium bis(trifluoromethanesulfonyl)imide (PYR14TFSI) data reported by Martinelli et al [35] is given for comparison.

Figure 2 shows ionic conductivity (σ) values of the TPE and PEO-LiTFSI on heating from −50 °C to 90 °C. Ionic conductivity data of PYR_14_TFSI [35] shown in Figure 2 demonstrates its higher conductivity comparison with both solid electrolytes. The σ of TPE increases up to values close to 10^−2^ S·cm^−1^, and likewise decreases on going from 90 °C to 25 °C, where it attains a value of 5 × 10^−4^ S·cm^−1^. As a consequence of the very low fraction of TPE suffering phase transitions in the temperature range studied, the heating and the cooling cycle measurements produce the same σ values, and so also with regards to σ variation with temperature, the TPE can be considered as a liquid. On the contrary, PEO-LiTFSI suffers the melting of the crystalline phase at about 50 °C on heating, and on cooling, an abrupt decrease of σ is seen below 50 °C, caused by the crystallization of PEO. Hence, the cooling and heating scans do not coincide in the vicinity of the phase transition. As a consequence, under 50 °C, the difference in σ between the TPE and PEO-LiTFSI becomes progressively higher.

**Figure 3:**
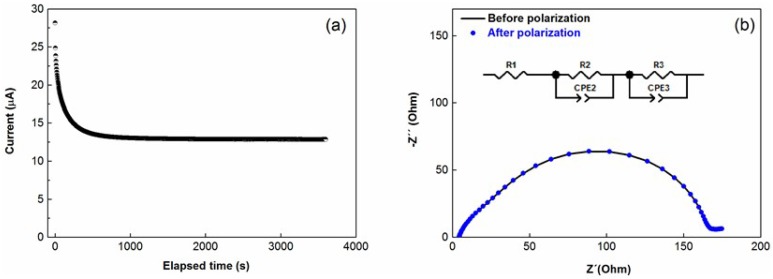
(**a**) Chronoamperometry of the Li/TPE/Li cell; (**b**) the alternative current (AC) impedance spectra before and after polarization. Inset (**b**): the equivalent circuit used for the fitting of the spectra, *R*_1_ corresponds to *R*_o_, *R*_ss_ is the sum of *R*_2_ and *R*_3_ to *R*_Co_ and *R*_Css_. Test was performed at 60 °C.

The $t_{{\mathrm{Li}}^{+}}$ is a very important characteristic of an electrolyte. A higher $t_{{\mathrm{Li}}^{+}}$ can reduce concentration polarization during charge/discharge steps and, consequently, can increase power density. Moreover, it can hinder Li metal dendrite growth and avoid decomposition and precipitation of the lithium salt. Figure 3a depicts the chronoamperometry of the symmetrical Li-Li coin cell with the investigated TPE. The AC impedance spectra before and after polarization of the cell are exhibited in Figure 3b. The equivalent circuit used for the determination of *R*_o_, *R*_ss_, *R*_Co_ and *R*_Css_ values is shown as an inset in Figure 3b.

Lithium ion transference number of the investigated TPE with EO/Li ratio 12, as obtained from Equation (2), is 0.08 ± 0.01 at 60 °C, which is lower than the value obtained for PEO-LiTFSI (EO/Li ~20), which is 0.25 ± 0.01. Such a difference is the direct consequence of the lower molar fraction of Li cations in the TPE ($\chi_{{\mathrm{Li}}^{+}}$ = 0.17) than in PEO-LiTFSI ($\chi_{{\mathrm{Li}}^{+}}$ = 0.5) or, in other words, while in the reference electrolyte, 50% of the ionic species are Li cations, the amount of Li cations in the TPE is only the 17% of the ionic species. A relatively low transport number ($t_{{\mathrm{Li}}^{+}}$) of TPE does not mean lower lithium ion conductivity ($\sigma_{{\mathrm{Li}}^{+}}$) in comparison with PEO-LiTFSI. In particular, as can be seen in Table 2, TPE has 1.9 times higher Li-ion conductivity compared with the reference at 60 °C. Moreover, we anticipate that the difference will increase over an order of magnitude at temperature below 60 °C, when the reference electrolyte will be partly crystalline because of PEO, while the TPE will remain amorphous. 

**Figure 4:**
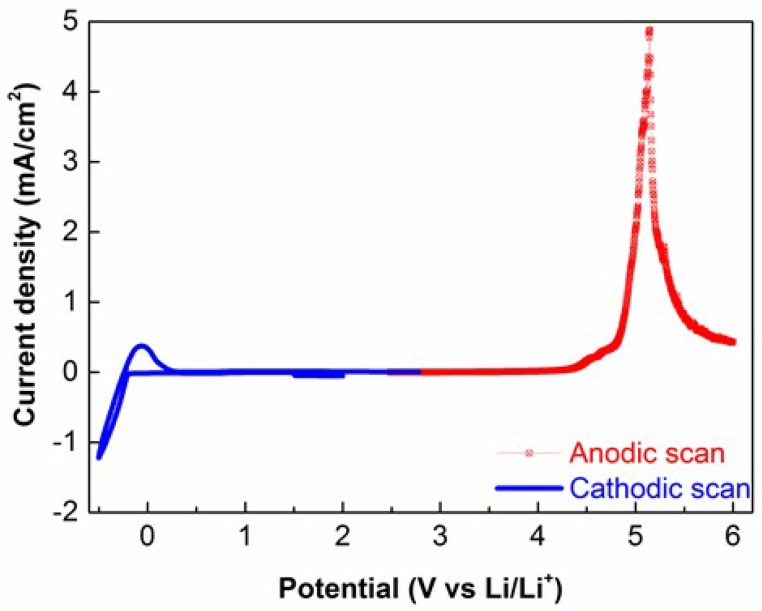
Cyclic voltammetry (CV) (blue) and linear sweep voltammetry (LSV) (red) curves of the TPE measured at 60 °C.

The electrochemical stability of the electrolyte is a fundamental property that determines the electrochemical behavior of the whole solid state battery. Figure 4 shows the electrochemical stability of the TPE under study towards anodic oxidation and cathodic reduction reactions. From the cathodic profile, reversible lithium plating and stripping processes are well evidenced. On the other hand, anodic LSV scab showed that the investigated electrolyte is anodically stable up to 4.2 V, which is a typical value for PEO based solid electrolytes.

**Figure 5:**
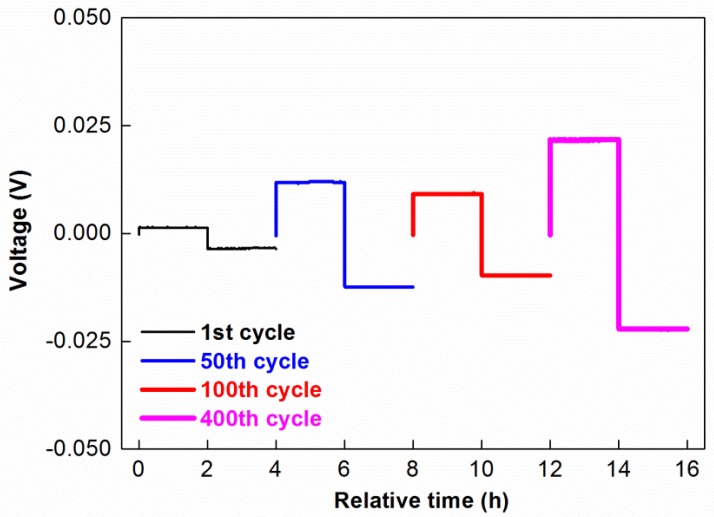
Cell voltage versus test time of lithium striping-plating in a symmetrical coin cell Li/TPE/Li measured on 1st, 50th, 100th, and 400th cycles. Cycling conditions: 60 °C, current density: 1 mA·cm^−2^, duration of each step 2 h.

Lithium metal electrode at contact with unappropriated solid electrolyte may show quite poor electrochemical behaviour (low coulombic efficiency, poor reversibility, and even lithium dendrite growth) due to cycling conditions (temperature, current density, depth of cycling) and properties of solid electrolyte layer (SEI) formed at lithium/solid electrolyte interface (nature, thickness, resistance etc.). Therefore, in this work, the compatibility of the TPE with the Li metal anode was evaluated by a galvanostatic stripping-plating test performed in Li-Li symmetrical coin cell with cycling conditions (temperature, current density, and depth of cycling) similar with full solid state cell application. Striping-plating curves for several separated cycles shown in Figure 5 with the aim of highlighting their evolution during the test. The TPE demonstrated quite low polarization and long term cyclability during more than 400 cycles (>1600 h) under relatively harsh cycling conditions. This result demonstrates that this solid electrolyte is well compatible with lithium metal anode that is necessary requirement for its further application in lithium metal solid state batteries.

**Figure 6:**
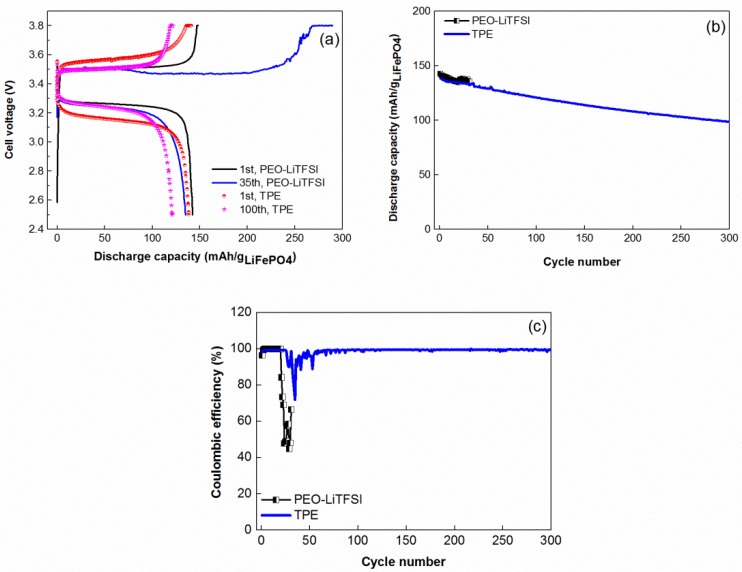
Electrochemical performance of solid-state coin cells Li-LiFePO_4_: (**a**) charge-discharge curves; (**b**) specific discharge capacity; and (**c**) coulombic efficiency versus cycle number. Cycling conditions: 60 °C, Constant Current-Constant Voltage (CCCV) charge at 0.2 C (charge current cut off 0.1 C), discharge at 0.5 C, cycling interval 2.5–3.8 V, positive electrode loading 0.5 mAh·cm^−2^.

Finally, the TPE was tested at 60 °C in all-solid-state coin cell with lithium metal anode and composite LiFePO_4_ cathode. Figure 6a presents charge-discharge curves of solid state coin cells with PEO-LiTFSI and TPE solid electrolytes. On the first cycle, polarization of the cell based on TPE is slightly higher in comparison with reference PEO-LiTFSI. However, during following cycles, charge-discharge profiles of both cells became quite similar.

The cycling performance of the solid state cell with TPE and PEO-LiTFSI is shown in Figure 6b. As it can be observed, upon cycling, solid state coin cell with TPE demonstrated more stable and higher coulombic efficiency (Figure 6a,c) and, as a result, much better electrochemical performance compared with the cell based on the reference PEO-LiTFSI compound.

It should be noted that relatively fast capacity decay of the nonoptimized solid-state coin cell prototype with TPE electrolyte could be related to several reasons, such as solid electrolyte impurities; traces of water in the electrolyte and cathode; and possible restructurization of TPE, which cointans noticeable amount of PYR_14_TFSI ionic liquid [23]. We believe that further optimization of solid state cell prototype and assembly-formation procedures may significantly improve its electrochemical performance and durability.

Thus, our investigation demonstrated that the developed polymer/ionic liquid thermoplastic solid electrolyte is a promising candidate for further development of all-solid-state batteries with relatively lower environmental impact.

## 4. Conclusions

The polymer/ionic liquid thermoplastic solid electrolyte based on PEO, modified sepiolite (TPGS-S), LiTFSI, and PYR_14_TFSI ionic liquid was successfully prepared using a solvent-free extrusion method beneficial for low cost and environment-friendly solid-state battery mass production. The physical-chemical, electrical, and electrochemical properties of the developed solid electrolyte were comprehensively characterized.

The TPE presented a maximum conductivity 0.5 S·cm^−1^ at 60 °C. The LSV curve showed that the electrolyte is stable up to 4.2 V versus Li/Li^+^ and possesses an excellent compatibility with lithium metal electrode during more than 1600 hours cycling under comparatively harsh cycling conditions (1 mA·cm^−2^, 2 mAh·cm^−2^).

Finally, the developed solid electrolyte demonstrated a promising cycling performance in nonoptimized prototype of truly Li-LiFePO_4_ solid-state coin cell working under relatively higher charge/discharge C-rates (0.2 C/0.5 C). 

Thus, the reported solid electrolyte can be considered as a promising candidate for further development of solid-state batteries with charge cut-off voltage about 4.0 V.

## Figures and Tables

**Table 1:** Main features of the investigated solid electrolytes. PYR14TFSI—1-Butyl-1-methylpyrrolidinium bis(trifluoromethanesulfonyl)imide; LiTFSI—lithium bis(trifluoromethanesulfonyl)imide; PEO—poly(ethylene oxide); TPGS-S—surface modified sepiolite; DSC—differential scanning calorimetry; TPE—thermoplastic polymer electrolyte.

Solid Electrolyte	PYR14TFSI mol m^−3^	LiTFSI mol m^−3^	PEO mol m^−3^	TPGS-S wt %	DSC Χc/Tm (%/°C)	σ (25 °C) mS cm^−1^	σ (60 °C) mS cm^−1^
PEO-LiTFSI	0	892	20670	0	32/53	0.01	0.5
TPE	1577	790	9826	2.5	5/38	0.50	3.0

**Table 2:** Ionic conductivity values of the investigated solid electrolytes.

Solid Electrolyte	σ (60 °C) mS cm^−1^	$\sigma_{Li^{+}}$ (60 °C) ^a^ mS cm^−1^
PEO-LiTFSI	0.5	0.125
TPE	3.0	0.240

Note: ^a^ *t*^+^ calculated by combined alternative current (AC) impedance and direct current (DC) polarization measurements reported above.
